# Genomic distribution and polymorphism of G-quadruplex motifs occupying ovine promoters and enhancers

**DOI:** 10.1007/s00335-023-09988-x

**Published:** 2023-03-25

**Authors:** Georgios C. Stefos, Georgios Theodorou, Ioannis Politis

**Affiliations:** Laboratory of Animal Breeding and Husbandry, Department of Animal Science, Agricultural University of Athens, 75 Iera Odos, 118 55 Athens, Greece

## Abstract

G-quadruplexes are non-canonical DNA structures that are formed in regions with short runs of guanines. During the last decade they have gained considerable attention due to their involvement in basic cellular processes, linking them to several physiological processes and pathological conditions. Regulation of gene transcription is among the crucial roles that G-quadruplexes play in the cells. Several ways in which these structures affect transcription have been described, both negatively and positively. Recently, G-quadruplexes have been shown to be implicated in the three-dimensional rearrangement of the chromosomes that enables the interaction of enhancers and gene promoters during regulation of transcription. Sheep is a species for which almost no G-quadruplex-related studies have been conducted and thus research on this species is kept out from the progress that has been made in the G-quadruplex field. In this context, we investigated the DNA sequences with potential to form G-quadruplexes (G4-motifs) in the ovine enhancers and promoters. We describe the distribution of G4-motifs within the regulatory regions which is shown to be enriched in G4-motifs in a way similar to other mammals. Furthermore, our data suggest that G4-motifs promote promoter-enhancer interactions in sheep. The single nucleotide polymorphisms colocalizing with promoter- and enhancer-associated ovine G4-motifs constitute a considerable pool of polymorphism and given the crucial role of these specific G4-motifs on regulation of transcription, we suggest this polymorphism as an interesting target for ovine genetic studies.

**Supplementary Information:**

The online version contains supplementary material available at 10.1007/s00335-023-09988-x.

## Introduction

DNA does not solely exists in the right-handed Watson–Crick B-form, but can also be found in alternative structures like cruciforms, supercoiled DNA, triple stranded DNA, Z-DNA, and G-quadruplexes (G4s) (Wells et al. 2007). The latter are formed when short ranges of guanines (G-runs) form planar tetramers which are held by multiple Hoogsteen hydrogen bonds and stacked and stabilized by monovalent cations (Spiegel et al. 2020). The existence of the G-quadruplex structures has been proven by several experimental approaches, including deep sequencing-based methods (Stefos et al. 2019). Although it has already been concluded from the latter methods that G-quadruplexes can be formed on several sequence motifs, the motif (G_≥3_N_1-12_)_3_G_≥3_ is among the ones that have been mostly used in the literature. The DNA sequences that fit motifs which can potentially form G4-structures are called G4-motifs hereafter.

G-quadruplexes are known to play an important role in several key cellular processes like regulation of transcription, translation, replication, and telomere homeostasis (Varshney et al. 2020). Regarding regulation of transcription, the high density of G4s in promoters and specific transcribed regions of several species (Huppert and Balasubramanian 2007; Marsico et al. 2019; Saad et al. 2019; Cagirici and Sen 2020; Stefos et al. 2022), supports their significance. Indeed, several mechanisms through which G-quadruplexes act have been reported. Firstly, when they are located on the template strand of the transcripts, they have a negative role on transcription since they act as obstacles for the DNA polymerase. Secondly, when G-quadruplexes are found on the coding strand they can either facilitate transcription by maintaining an open chromatin statue or repress it through the formation of DNA:RNA hybrids. Lastly, when they are located on regulatory regions, they can either facilitate or hinder the recruitment of transcription factors (Varshney et al. 2020; Spiegel et al. 2021).

A rather new finding is that G4s located on regulatory regions, i.e., promoters and enhancers, can contribute in the transcription regulation via promoting enhancer/promoter interaction (Robinson et al. 2021). The G4-motifs located on these regulatory regions are called regulatory-G4-motifs hereafter. Although promoter regions carry the elements which are sufficient for the basal transcription, enhancers regulate transcription from (even significantly considerable) distance. This is facilitated by DNA looping that enables the distal interaction of regulatory domains (Schoenfelder and Fraser 2019). Recently, sizeable attention has been given to the role of G4s on this interaction (Robinson et al. 2021). Several models have been suggested for the implication of G4s in both the stabilization of the DNA loops and the recruitment of transcription-enhancing protein complexes (Hegyi 2015; Kuznetsov et al. 2018; Hou et al. 2019, 2021; Li et al. 2021; Lyu et al. 2022).

Although G-quadruplexes have been thoroughly investigated in human and other organisms, there is an obvious lack of similar studies in livestock animals in general (Stefos et al. 2019), and specifically in sheep. Given the significant function of G-quadruplexes in many basic cellular processes, biochemical and genetic studies employing ovine G-quadruplexes could contribute to the better understanding of the physiology of this species and the association of polymorphisms with certain traits of agricultural interest. Here, we describe the occupation of the ovine regulatory regions by G4-motifs, examine their role in enhancer-promoter interactions and point out the polymorphism of these specific motifs.

## Materials and methods

### Genomic data acquisition

The coordinates and the FASTA files of the promoter regions spanning 500 nt upstream the transcription start sites (TSSs) of the NCBI RefSeq genes (assembly: oviAri 4) were obtained using the UCSC Table browser tool. The coordinates and FASTA of the enhancer RNAs (eRNAs) (assembly: oviAri 3) were downloaded from the Animal-eRNAdb (Jin et al. 2022). The single nucleotide polymorphisms (SNPs) were downloaded from the iSheep database (Wang et al. 2021). Among the ~ 70 million SNPs of the database, SNPs called by whole genome sequencing on 355 animals are included, as well as SNPs from Illumina’s ovine 50 K and 600 K BeadChips. The list with the 922 SNPs that are associated with phenotypic traits according to 52 publications, was downloaded from the iSheep database (Wang et al. 2021). For conversions of coordinates between assemblies, the UCSC Genome Browser LiftOver tool was used.

### Identification of G4-motifs

The identification of G4-motifs on the FASTA files was done with the pqsfinder package in R (Hon et al. 2017). The algorithm was set to search for motifs having all G-runs longer than or equal to 3 nt, loops up to 12 nt, total length smaller than 110 bp and without mismatches.

### Ontology terms overrepresentation analysis

The list of genes that a) their promoters carry G4-motifs and b) are associated, according to Animal-eRNAdb, to eRNAs that also carry G4-motifs, was used as input for overrepresentation analysis of *Gene Ontology Biological Processes* (GOBP) terms in the online tool Panther (Mi et al. 2009), using *Bos taurus* as background organism. Next, all overrepresented terms with FDR < 10^–5^ were used as input in the REVIGO online tool (Supek et al. 2011) in order to remove redundant terms.

### SNP density analyses

SNP density or number of SNPs per base-pair (bp), of a given region was calculated by dividing the number of SNPs overlapping with this region by the total length of the region. For testing whether the SNP density of a given region differs from the background, the SNP density of this region was compared with that of random regions of the same length and the test was repeated as many times as described in the corresponding figure legend. When these comparisons were done for each chromosome specifically, the query and the random regions were from the same chromosome. The significances were defined as the ratios of the randomization tests that resulted to different (higher/lower) SNP density.

## Results and discussion

### G4-motifs on regulatory regions

The two regulatory features that are considered here are the promoter regions and the enhancer RNAs. As promoters, are defined the 500 nucleotides upstream the TSSs and are named prom500 hereafter. This rather short promoter length was chosen to minimize overlapping with transcribed regions of neighboring genes. The eRNAs were obtained from the Animal-eRNAdb and are 6 Kb long (Jin et al. 2022). The G4-motifs that are located on both features were identified with the pqsfinder algorithm.

The formula G_≥3_(G_≥3_N_1–12_G_≥3_N_1–12_G_≥3_)_≥3_ that was used for the G4-motif search was chosen over a formula with loops up to 7 nts (N_1–7_) in order to avoid missing the considerable fraction of those motifs with longer loops which are known to be present in promoter regions and overlap with experimentally identified G-quadruplex structures (Lago et al. 2021). Our search resulted in G4-motifs located on both DNA strands regarding the direction of the relative transcript and are not discriminated in the subsequent analyses.

**Fig. 1:**
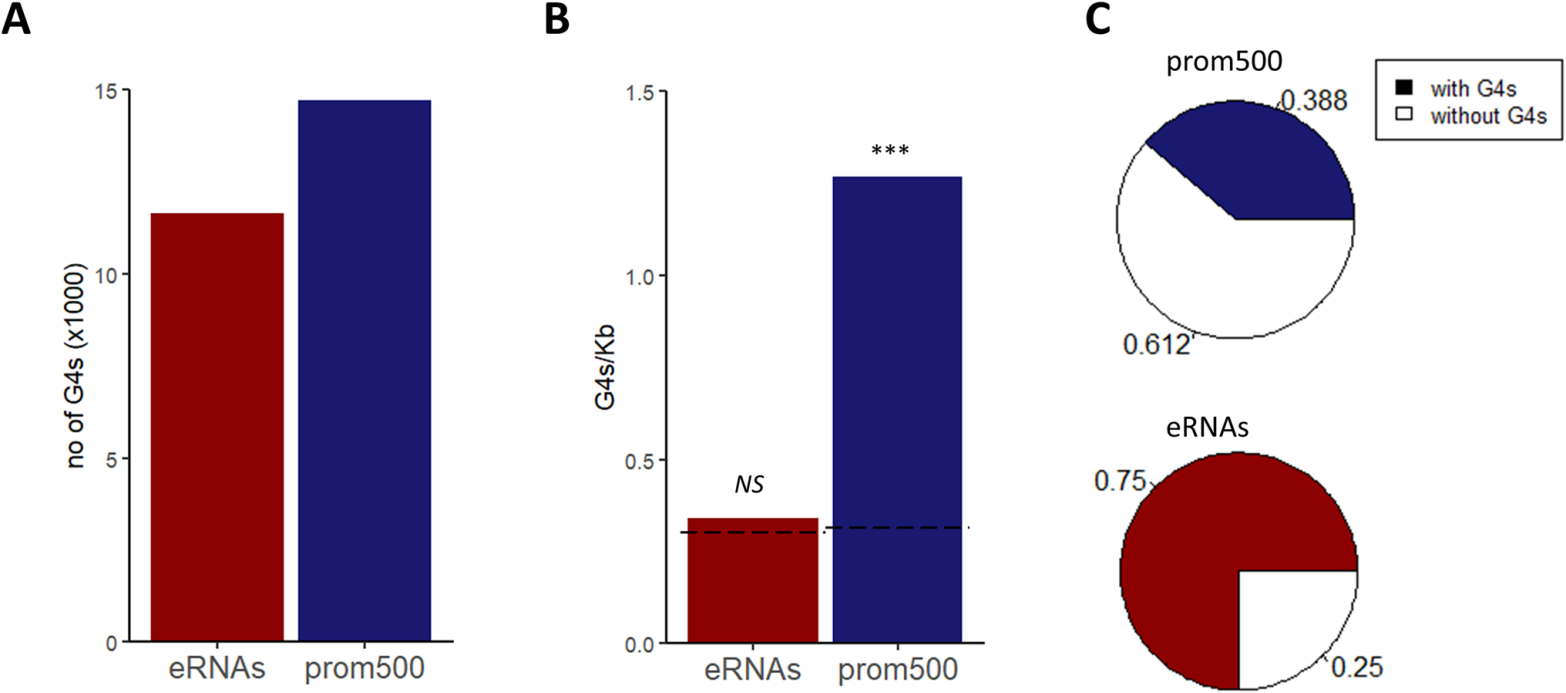
Distribution of G4-motifs on regulatory regions. **A** Numbers of G4-motifs located on prom500 and eRNAs. **B** Densities of G4-motifs on prom500 and eRNAs. Densities are expressed as number of G4-motifs per kilobase. Dashed lines show the background densities, defined as the mean of 500 randomization tests. For each test, 500 or 50 random sequences of the ovine genome, having the same length with prom500 and eRNAs, respectively, were subjected to calculation of their G4-motifs densities. Statistical significance was defined as the ratio of tests in which the mean value was higher/lower than the actual mean value of the feature. **C** Ratios of prom500 and eRNAs that have at least one G4-motif. *NS* not significant; ***P < 0.001

In Fig. 1A the numbers of identified G4-motifs in prom500 and eRNAs are shown. Although both features contain a considerable amount of G4-motifs, promoters show significantly higher density than the background (Fig. 1B). Concerning promoters, which are known to be enriched in G4-motifs in several species (Marsico et al. 2019), the density in sheep although higher than what is reported for human and cow (Huppert and Balasubramanian 2007; Stefos et al. 2022), is in the same range with these species. This density difference is expected because here are reported also those motifs with loop lengths 8–12 nt that are not considered in the other species. Figure 1C shows that while the 75% of the eRNAs have at least one G4-motif, the latter are present only in around 40% of the prom500. This considerable difference between the two features was rather expected since the length of eRNAs was 6 Kb while this of prom500 was 0.5 Kb. Supplementary Fig. 1 shows that the distribution of G4-motifs on ovine promoters is in line with what has been reported for other mammals (Verma et al. 2008; Gong et al. 2021); the occurrence of G4-motifs is higher closer to the transcription start site. On the contrary, eRNAs do not show any particular distribution pattern (data not shown).

**Fig. 2:**
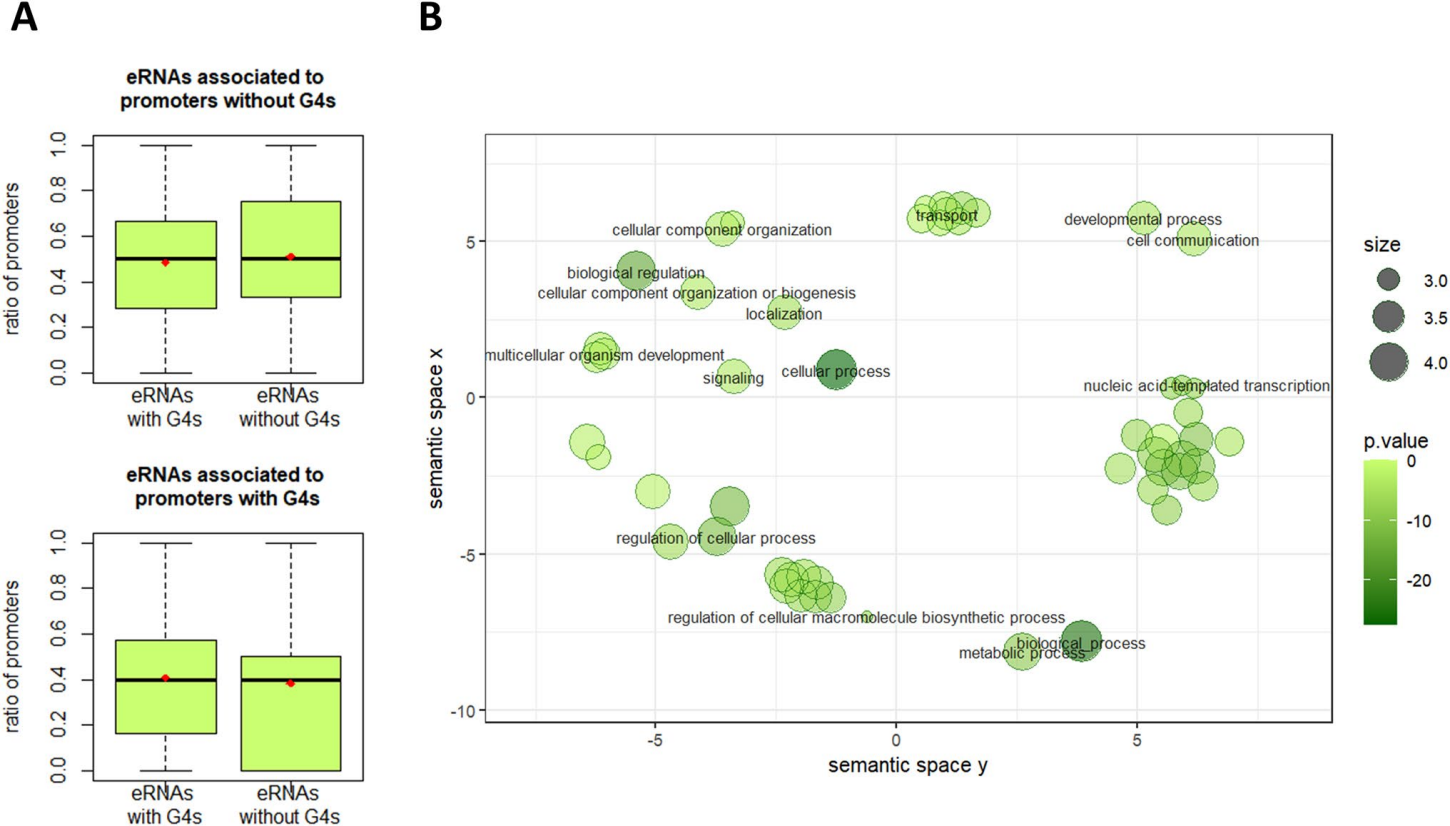
G4-motifs in enhancers-promoters interaction. **A** Each eRNA is associated to one or more genes. Boxes show the ratios of promoters of the genes that are associated to eRNAs, with or without G4-motifs. The associations between genes (promoters) and eRNAs were obtained by the Animal-eRNAdb. Red dots show average values. For both plots the difference between eRNAs with G4-motifs and eRNAs without G4-motifs is statistically significant (*P* < 0.05, Wilcoxon test). **B** GOBP overrepresented terms in genes that have promoters carrying G4-motifs and are associated with eRNAs carrying G4-motifs. The color of each dot represents the adjusted *p* value of the term, given as the negative log value, while the size represents the log of the number of annotations

Given the current notion that G4s promote the interaction between enhancers and promoters, in this way regulating transcription (Lyu et al. 2022; Dumas et al. 2021; Robinson et al. 2021; Hegyi 2015), next, we tried to investigate this scenario in sheep. Thus, both prom500 and eRNAs were first assigned to two categories according to whether they carry G4-motifs or not and then all combinations of interactions between eRNAs and prom500, as defined by animal-eRNAdb, were counted. Figure 2A shows that prom500 carrying G4-motifs interact more often with eRNAs which also carry G4-motifs than with eRNAs that do not carry G4-motifs (*lower panel*). On the contrary, prom500 that do not carry G4-motifs interact more often with eRNAs that also do not carry G4-motifs than with eRNAs carrying G4-motifs (*upper panel*). Although Fig. 2A does not provide any solid evidence for a G4-mediated enhancer-promoter interaction in sheep, our data are in line with what has been shown in other organisms, for which more sophisticated approaches, that provide direct evidence for 3D chromatin-chromatin interactions, have been used.

Figure 2B shows the enrichment of the non-redundant Gene Ontology terms (see “Materials and methods”) among all genes that both (a) carry G4-motifs on their prom500 regions, and (b) are associated with eRNAs which also carry G4-motifs. Among the enriched terms, there are some which are related to processes with special interest for livestock animals, like development and metabolism. Taken together, the indications that the ovine G4-motifs may shape the transcriptional program through chromatin rearrangement and that the genes implicated in this process seem to be important in sheep breeding, show that the role of regulatory G4-motifs in transcriptional regulation might be worth further investigation in this species.

### Polymorphism of G4-motifs located on regulatory regions

**Fig. 3:**
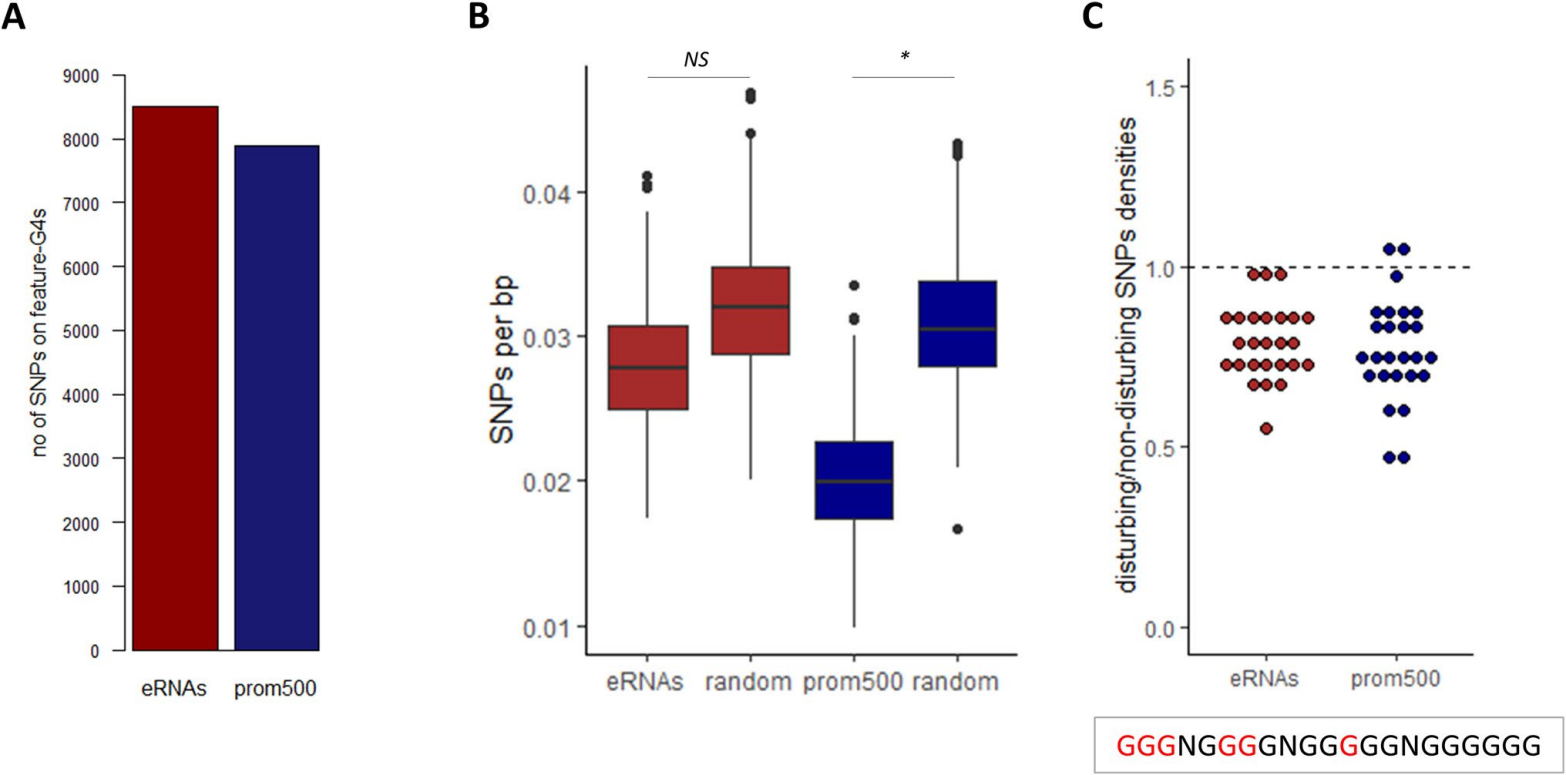
SNPs on regulatory G4s-motifs. **A** Number of SNPs colocalizing with G4-motifs of eRNAs and promoters. **B** SNPs densities of prom500, eRNAs, and random ovine sequences. For each feature, the SNPs density of 100 random feature-specific G4-motifs was compared with the density of 100 random sequences of comparable length and GC content, derived from the ovine genome. The randomization tests were repeated 500 times and the significances were defined as the ratio of random tests for which random sequences were higher than random G4-motifs. **C** Upper panel: Ratios of the densities of disrupting to densities of non- disrupting SNPs of the G4-motifs which carry four G-runs and are located on prom500 and eRNAs. Each dot represents the ratio in one chromosome. The dashed line shows ratio equal to 1. Lower panel: Disruptive (red) and non-disruptive (black) nucleotides on an example of G4-motif. As disruptive are defined all the Gs of 3-nt-long G-runs, the second and third Gs of 4-nt-long G-runs and the middle G of 5-nt-long-G-runs. Gs located on G-runs longer than 5 nucleotides are considered as non-disruptive (Nakken et al. 2009). *NS* not significant; *P < 0.05

In order to examine the polymorphism of G4-motifs located on prom500 and eRNAs, SNPs obtained from iSheep were used (Wang et al. 2021). As it is shown in Fig. 3A, there is a considerable number of SNPs overlapping with G4-motifs which are located on eRNAs and promoters. The overall SNPs densities for both features is presented in Fig. 3B. G4-motifs located on prom500 are less polymorphic than the background, while this difference is not significant for the G4-motifs located on eRNAs. It should be noted that in order to examine solely the polymorphism of the G4-motifs per se, in Fig. 3B the polymorphism associated to their GC-content was filtered out by using random sequences with similar GC-content. Next, we assessed the polymorphism within the different parts of G4-motifs. For this, we defined as disruptive nucleotides all those on which the occurrence of SNPs could result in disruption of the G4 structure (Nakken et al. 2009) (Fig. 3B-*lower panel*). It should be noted that for assigning disruptive nucleotides, only G4-motifs with 4 G-runs were taken into consideration since the presence of extra G-runs can serve as “spare tires” (Fleming et al. 2015), neutralizing the effect of disruptive nucleotides. Figure 3C (*upper panel*) shows that disruptive nucleotides are less polymorphic than non-disruptive ones. This finding is in line with what has been described in other mammals (Nakken et al. 2009; Stefos et al. 2022) and is an indication suggesting functional importance of G4 structures in eRNAs and promoters.

**Fig. 4:**
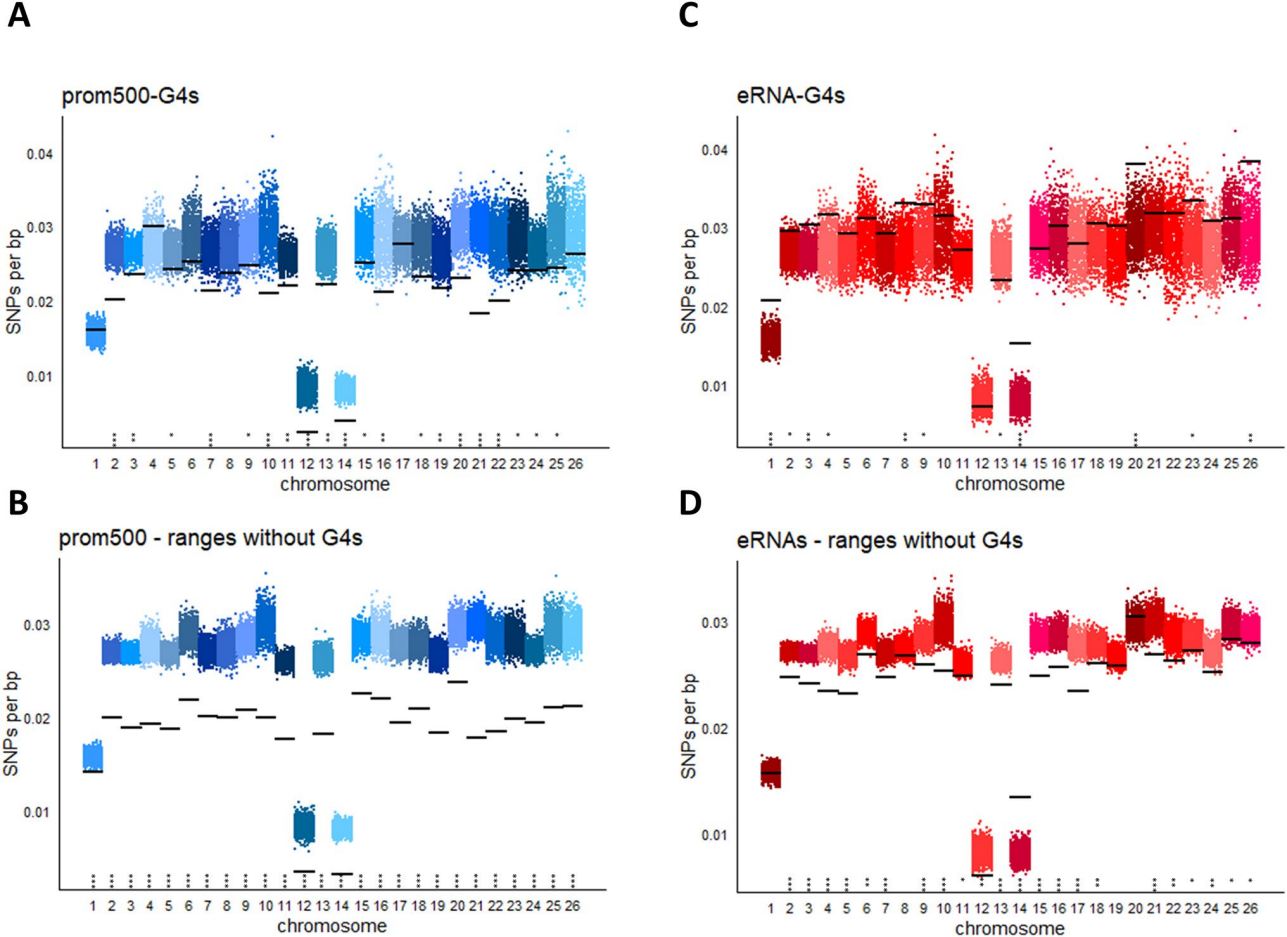
SNP densities on regulatory regions. **A**–**D** SNP densities on eRNAs and prom500 for all chromosomes. SNP densities on regulatory G4-motifs (**A**, **C**) or on the ranges of eRNAs and prom500 that are free of G4-motifs (**B**, **D**) are shown by horizontal black lines. Blue/red dots (10,000 per chromosome) show the SNP densities of 10,000 randomization tests. In each test, SNP densities of random fragments of equal number and length were calculated. Stars above chromosome numbers indicate that the black lines are significantly higher/lower than the random tests. Significances were defined by the ratio of the random tests with higher/lower SNP density than that corresponding to black lines, as described in materials and methods. *NS* not significant; *P < 0.05; **P < 0.01; ***P < 0.001

For a deeper analysis of the polymorphism of regulatory G4-motifs, their SNPs densities as well as the densities of their context fragments were calculated thoroughly. Starting with G4-motifs located on prom500, as shown in Fig. 4A, most of them are significantly less polymorphic than in the background. However, the prom500 regions that do not bear G4-motifs are even less polymorphic (Fig. 4B). The situation is rather different for the eRNAs (Fig. 4B, C). Although these features are still less polymorphic compared to the background, the eRNA-G4-motifs do not significantly differ. It is of interest that in some cases eRNA-G4-motifs appear to have even higher SNP density than the background. Concerning the polymorphism of promoters and enhancers per se, there are studies in several species with diverse results. For example in human, Drake et al. (2006) mention that promoters and enhancers, in the non-conserved genomic regions (that make up the biggest part of the genome), as defined by ChromHMM, have significantly lower SNP density than the background. On the other hand, Telenti et al. (2016) considering the SNPs from 10,000 human genomes report promoters to have higher SNPs density than the background while enhancers have lower. These dissimilarities may arise from several factors, like the pool of SNPs that were used, the way that the genomic features were defined, differences in the algorithms that were used, the inclusion or not of the repetitive genome etc. Our study shows that the relation of SNP density in ovine regulatory regions compared to the genome is closer to what is reported by Drake et al. (2006) in humans.

**Fig. 5:**
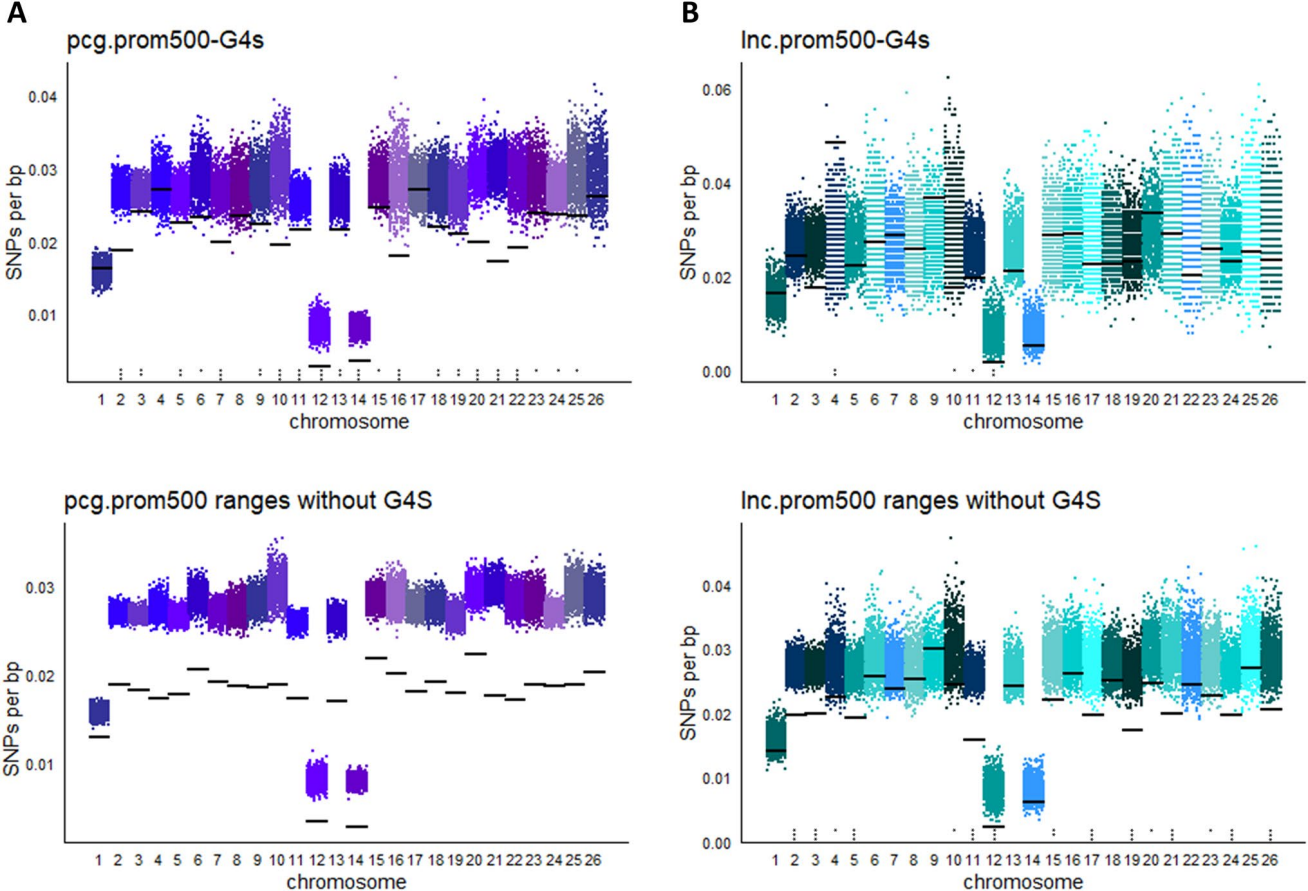
SNP densities on prom500 regions. **A** SNP densities of prom500 of proteins coding genes for all chromosomes: By horizontal black lines are shown the SNP densities of prom500 G4-motifs (upper panel) or the SNP densities of the ranges of prom500 that are free of G4-motifs (lower panel). Colored dots (10,000 per chromosome) show the results of 10,000 randomization tests in which SNP densities of random fragments of equal number and length were calculated. Stars above chromosome numbers indicate that the black lines are significantly higher/lower than the random tests. **B** SNP densities as described for **A**, but for lncRNAs. Significances were defined by the ratio of the randomization tests with higher/lower SNP density than that corresponding to black lines, as described in materials and methods. *NS* not significant; *P < 0.05; **P < 0.01; ***P < 0.001

Next the prom500 regions were studied in more detail, examining the regulatory regions of genes encoding for proteins and those corresponding to long non-coding RNAs (lncRNAs) (Fig. 5). The polymorphism in promoters of protein coding genes, as expected since they constitute the majority of genes, is similar to that of all promoters (Fig. 5A). On the other hand, promoters of lncRNAs, are more polymorphic, while the G4-motifs located on these promoters do not significantly differ from the background overall (Fig. 5B). Concerning the polymorphism of promoters regions per se in livestock animals, similar results to those shown in Fig. 5B have also been reported in other studies. In particular, swine protein coding promoters are less polymorphic than lncRNA promoters and the latter are slightly less polymorphic than random regions (Yang et al. 2017). In a study concerning bovine long intervening non-coding RNAs (lincRNA) (Huang et al. 2012), the upstream regions of lincRNAs are shown to be more polymorphic than those of protein coding genes. Although the study examines lincRNAs and not lncRNAs, the message remains that the upstream regions of protein coding genes carry less SNPs compared to those of non-coding genes.

The fact that G4-motifs in sheep carry more SNPs compared to their contextual features is not in line with what has been shown by Nakken et al. (2009) for humans. However, there are two major methodological differences between the two studies. Firstly, in the study of Nakken et al*.* the regions around TSSs (downstream and upstream) have been considered, while in the present study only the 500 bp upstream of the TSSs. Secondly, Nakken et al*.* compared the G4-motifs with random sequences of the same GC-content in an attempt to investigate the mechanism of G4-motifs mutagenicity. Since the scope of the present study was to investigate the degree of polymorphism of the regulatory regions found on G4-motifs, the correction for GC-content would be rather misleading.

The data of the present study show that the G4-motifs located on both ovine promoters and enhancers, consist a considerable pool of polymorphism within regulatory regions. The above conclusion along with (a) the effect that the regulatory regions-associated SNPs have on gene expression (Mattioli et al. 2019) and (b) the connection of SNPs on regulatory G4-motifs with physiological and pathological processes (Gong et al. 2021; Hou et al. 2021), render the polymorphism of the regulatory regions-G4-motifs an interesting resource for genetic studies in sheep.

### Regulatory G4-motifs and genetic studies

Given the significant role that polymorphisms on the G4-motifs of the regulatory regions can play on gene expression, the distribution of SNPs on these DNA regions was further examined (Table 1). Although, more than 26,000 G4-motifs are found on both regulatory elements, none of the 922 SNPs that are associated with phenotypic traits which obtained from the iSheep database (Wang et al. 2021), overlap with these G4-motifs. These SNPs have resulted from genome-wide association studies (GWAS) and are manually curated from 52 publications by iSheep. Next, to predict whether future GWAS analyses can reveal any role for the polymorphism of the regulatory G4-motifs, by using some of the present tools, the information regarding regulatory G4-motifs of the most used commercial SNP arrays was investigated. As shown in Table 1, Illumina’s OvineSNP50 BeadChip contains only 7 SNPs overlapping with regulatory G4-motifs, while the high density SNP array (600 K) contains 71 overlapping SNPs. Taken together, the above show that although there is a considerable degree of polymorphism within the regulatory G4-motifs which would be expected to significantly affect gene expression, tools that are commonly used in the genetic studies in sheep may not be enough on their own to reveal the association of these regions with phenotypic traits of agricultural importance.

**Table 1:** SNPs of commercial genotyping arrays or associated with QTLs overlapping with regulatory G4-motifs

	Total	Overlapping with eRNA G4-motifs	Overlapping with prom500 G4-motifs
G4-motifs		11665	14724
SNPs associated with QTLs	922	0	0
SNPs on the 600 K array	606006	43	28
SNPs on the 50 K array	51132	5	2

## Conclusion

Our analysis reveals that, similarly to other organisms, the ovine promoters and enhancers carry an increased amount of G4-motifs. These motifs have higher SNP loads compared to their context features, which renders them a considerable pool of polymorphism within regulatory regions. This, given the significant role that regulatory-G4-motifs have on gene expression, could make them attractive targets for genomic studies. However, present tools that are commonly used in sheep genetics, although they have contributed to a significant progress in the field, cannot support the discovery of associations between regulatory-G4-motifs and phenotypic traits. Whole genome sequencing can be a much more effective, but rather costly, approach in this direction. Alternatively, focusing on the polymorphisms of these specific regions could be a beneficial strategy for sheep genetics. Among the technologies that could be used in that regard, custom genotyping strategies are the most advanced and their cost is being constantly reduced. The incorporation of such approaches in sheep genetics could be beneficial for the association of G4-motifs polymorphism with important traits for sheep breeding.

## Supplementary Information

Below is the link to the electronic supplementary material.

Supplementary file1 (PDF 96 kb)
