# Supplementary material for: Genomic distribution and polymorphism of G-quadruplex motifs occupying ovine promoters and enhancers
Source: Mamm Genome. 2023 Mar 25;34(3):473–81. doi: 10.1007/s00335-023-09988-x (PMC10382345; doi:10.1007/s00335-023-09988-x)
Supplement: Supplementary file 1 — Supplementary file1 (PDF 96 kb) [file 335_2023_9988_MOESM1_ESM.pdf]

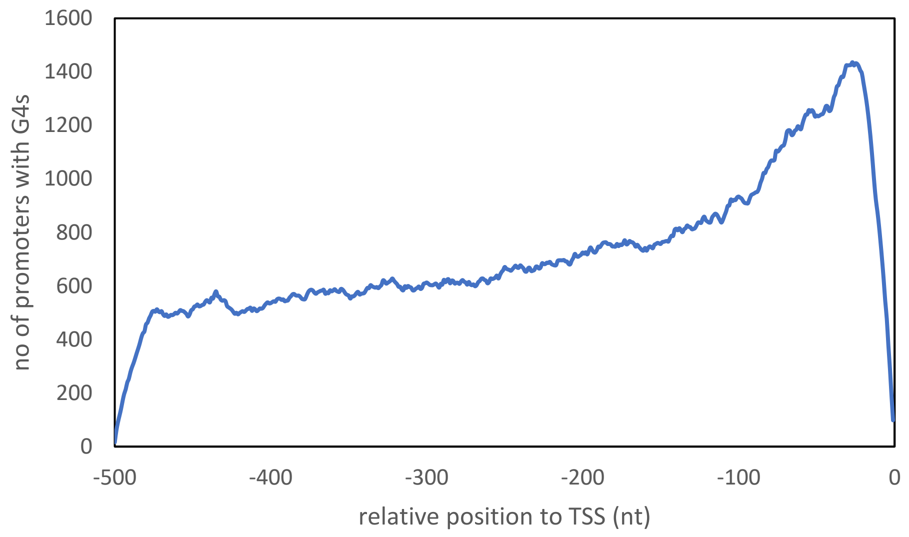

Supplementary Figure 1:

Aggregate plot for the occurrence of G4-motifs on the region spanning 500 nucleotides upstream the transcription starts sites (TSSs) of the ovine genes.

The depressions at the ends of the plot are due to the fact that only the G4-motifs falling entirely within the plotted region have been assigned to the extreme nucleotides. Those G4-motifs that partly fall within this region (and do contain nucleotides from this region) are ignored. Thus, the depression near TSSs cannot be entirely attributed to an occurrence of less G4-motifs on the TSSs like is shown for other mammals (Verma et al. 2008; Gong et al. 2019).
